# Clinical significance of histologic chorioamnionitis with a negative amniotic fluid culture in patients with preterm labor and premature membrane rupture

**DOI:** 10.1371/journal.pone.0173312

**Published:** 2017-03-09

**Authors:** Jeong Woo Park, Kyo Hoon Park, Eun Young Jung

**Affiliations:** 1 Department of Obstetrics and Gynecology, Seoul National University College of Medicine, Seoul, Korea; 2 Department of Obstetrics and Gynecology, Inje University College of Medicine, Ilsan-Paik Hospital, Gyeonggi, Korea; 3 Department of Obstetrics and Gynecology, Seoul National University Bundang Hospital, Seongnam, Korea; BC Children's Hospital, CANADA

## Abstract

**Objective:**

To evaluate the effect of histological chorioamnionitis (HCA) with a negative amniotic fluid (AF) culture on adverse pregnancy and neonatal outcomes and inflammatory status in the AF compartment in women with preterm labor or preterm premature rupture of membranes (PPROM).

**Methods:**

This is a retrospective cohort study of 153 women diagnosed as having a preterm labor or PPROM (20–34 weeks) who delivered singleton gestations within 48 hours of amniocentesis. AF obtained through amniocentesis was cultured, and interleukin (IL)-6, IL-8, and metalloproteinase-9 (MMP-9) levels were determined. The placentas were examined histologically.

**Results:**

The prevalence of HCA with negative AF culture was 23.5% (36/153). The women with HCA but with a negative AF culture (group 2) and those with a positive AF culture (group 3) had a significantly lower mean gestational age at amniocentesis and delivery than those with a negative AF culture and without HCA (group 1). Women in group 3 had the highest levels of AF IL-6, IL-8, and MMP-9, followed by those in group 2, and those in group 1. Composite neonatal morbidity was significantly higher in groups 2 and 3 than in group 1, but this was no longer significant after adjusting for confounders caused mainly by the impact of gestational age.

**Discussion:**

In the women who delivered preterm neonates, HCA with a negative AF culture was associated with increased risks of preterm birth, intense intra-amniotic inflammatory response, and prematurity-associated composite neonatal morbidity, and its risks are similar to the risk posed by positive AF culture.

## Introduction

Histologic chorioamnionitis (HCA) complicates as many as 30–80% of preterm births with preterm labor or premature rupture of membranes (PPROM), and has been shown to be a risk factor of adverse maternal and neonatal outcomes, including earlier gestational age at delivery and neonatal brain and lung injuries [1–4]. Moreover, HCA is associated with the presence of microbial invasion of the amniotic cavity (MIAC), serving as a route for ascending infection, when detected in preterm placenta [5–7].

Most of the previous studies that demonstrated a strong association between HCA and documented poor neonatal and pregnancy outcomes included mixed cases of HCA with and HCA without MIAC [2, 4, 7, 8]. However, the maternal and neonatal outcomes of women with HCA alone may differ from those of women who had both MIAC and HCA because MIAC is the advanced stage of ascending intrauterine infection, and infection and inflammation with microorganisms in the amniotic fluid (AF) may result in direct invasion of the fetus, contributing to clinical disease in the neonatal period [3, 6]. By contrast, HCA alone is the early stage (localized inflammatory processes detected in the membranes), as proposed by Romero et al [3, 6]. Nevertheless, to date, to what extent or whether HCA alone may have adverse effects on maternal and neonatal outcomes and intense inflammatory responses in the AF is unclear. Indeed, a substantial number of women with preterm labor or PPROM have HCA but have negative AF culture results [5, 7, 9, 10]. The purpose of this study was to determine the frequency and impact of HCA with a negative AF culture on adverse pregnancy and neonatal outcomes, and inflammatory status in the AF compartment in women with preterm labor or PPROM.

## Materials and methods

This is a retrospective cohort study conducted at Seoul National University Bundang Hospital (Seongnam, South Korea) from June 2004 to August 2013. The study population consisted of consecutive women diagnosed as having either preterm labor and intact membranes or PPROM who underwent transabdominal amniocentesis. The patients were retrospectively identified by searching our perinatal database according to the following criteria: (1) singleton gestation; (2) amniocentesis performed; (3) delivery at a gestational age between 20+0 and 34+6 weeks; (4) delivery within 48 hours of amniocentesis; and (5) histopathological examination of the placenta. The exclusion criteria were as follows: (1) multiple gestations; (2) a time interval of >48 hours from amniocentesis to delivery (used to maintain a meaningful temporal relationship between the AF culture and the placental histological examination results); and (3) major congenital anomalies. The data of 58 patients were previously reported in a previous retrospective study that evaluated the predictive value of intra-amniotic and serum markers for inflammatory lesions of preterm placenta [7]. The diagnostic criteria for preterm labor and PPROM were previously described in detail elsewhere [11, 12]. The primary outcome measures were composite neonatal morbidity and mortality, inflammatory status in the AF, and the gestational age at which the clinical symptom and delivery occurred. The local ethics committee of Seoul National University Bundang Hospital (project No. B-1105/128-102) approved this study. All the women provided written informed consent to undergo the amniocentesis procedure and for the use of AF samples for research purposes prior to the amniocentesis.

In women who were admitted under the diagnosis of either preterm labor or PPROM, and delivered a preterm neonate at our institution, amniocentesis for the assessment of microbiological and inflammatory statuses of the amniotic cavity was recommended. In addition, placentas were routinely sent for histopathological examination. Based on the results of the placental histological examination and AF culture, the subjects were divided into 3 patient groups as follows: (1) no placental inflammation with a negative AF culture result (group 1, n = 64), (2) placental chorioamnionitis with a negative AF culture result (group 2, n = 36), and a positive AF culture result (group 3, n = 53).

AF was aseptically obtained by performing abdominal amniocentesis under sonographic guidance. AF was analyzed for white blood cell (WBC) counts and cultured for aerobic and anaerobic bacteria, as well as genital mycoplasmas (*Mycoplasma hominis* and *Ureaplasma* species), according to previously described methods [11]. The remaining AF that was not required for clinical assessment was centrifuged at 1,500*g* and 4°C for 10 minutes. The supernatant was aliquoted and stored at −70°C until assayed. The samples were not subjected to freeze-thaw cycles before assay. The levels of interleukin (IL)-6, IL-8, and metalloproteinase-9 (MMP-9) in stored AF were measured by using an enzyme-linked immunosorbent assay human DuoSet Kit (R&D System, Minneapolis, MN, USA). The ranges of the IL-6, IL-8, and MMP-9 standard curves were 7.8–600, 31.2–2000, and 31.2–2000 pg/ml, respectively. All the samples were assayed in duplicate. The intra- and inter-assay coefficients of variation were each <10%. The AF culture results and WBC counts, but not AF IL-6, IL-8, and MMP-9 levels, were available to the managing obstetricians and neonatologists. Immediately after amniocentesis, WBC count and C-reactive protein (CRP) level in maternal blood were measured, and details of these measurements were previously described [11].

Management of preterm labor and PPROM, and clinical chorioamnionitis has been previously described in detail elsewhere [11, 12]. MIAC was defined as the presence of microorganisms in an AF culture. Acute histological chorioamnionitis was diagnosed when acute inflammation was observed in any placental tissue samples (amnion, chorion-decidua, umbilical cord, and chorionic plate). The presence of acute inflammation was noted and classified as grade 1 or 2 (Table 1) according to previously published criteria [10]. Funisitis was diagnosed when neutrophil infiltration was observed in the umbilical vessel walls or Wharton’s jelly. Intra-amniotic inflammation was defined as elevated AF levels of IL-6 and/or IL-8 (≥1.5 and/or ≥1.3 ng/mL, respectively) [13]. Clinical chorioamnionitis was diagnosed based on the criteria proposed by Gibbs et al [14]. Early-onset neonatal sepsis, respiratory distress syndrome (RDS), bronchopulmonary dysplasia (BPD), intraventricular hemorrhage (IVH) of grade II or higher, periventricular leukomalacia (PVL), and necrotizing enterocolitis (NEC) were diagnosed according to the definitions previously described in detail.[15] Composite morbidity was defined as the presence of any of these complications.

**Table 1:** Histological grade for acute intrauterine inflammation.

Amnion and chorion-decidua
Grade 1: at least one focus of > 5 neutrophils
Grade 2: diffuse neutrophilic infiltration
Umbilical cord
Grade 1: neutrophilic infiltration confined to umbilical vessel wall
Grade 2: extension of neutrophilic infiltration into Wharton’s jelly
Chorionic plate
Grade 1: > 1 focus of at least 10 neutrophilic collections or diffuse inflammation in subchorionic plate
Grade 2: diffuse and dense inflammation, neutrophilic infiltration into connective tissue of placental plate, or placental vasculitis

Continuous data are presented as mean ± standard deviation (SD); and dichotomous data, as frequency and associated percentage. Shapiro-Wilk and Kolmogorov-Smirnov tests were used to evaluate whether the data are normally distributed. As the continuous data were not normally distributed, non-parametric tests were used for the analyses. The Kruskal-Wallis test was used to test for comparison of continuous data among 3 groups. Multiple comparisons between the groups were performed by using the Mann-Whitey *U* test for continuous data and the *χ*^2^ or Fisher exact test for categorical data, adjusted with Bonferroni correction (P*<*0.0167). Multivariable logistic regression analysis was used to examine the relationship between the HCA with a negative AF culture, neonatal outcome, and inflammatory status in AF after adjusting for compounding factors (the effect of gestational age and fetal membrane status). All statistical analyses were performed by using SPSS version 20.0 for Windows (IBM SPSS Statistics, Chicago, IL, USA), and P values of <0.05 were considered statistically significant except in the pairwise comparison.

## Results

During the study period, 159 consecutive women with preterm labor (n = 83) and PPROM (n = 76) who met the inclusion criteria were identified. Of the 159 women, 5 had no available AF samples for retrospective analysis of IL-6, IL-8, and MMP-9 levels and one had no available placental pathological report. Thus, 153 women (95%) were included in the final analysis. Among the 153 women, 50% (76/153) had HCA and 20% (31/153) had funisitis. The prevalence of a positive AF culture was 35% (53/153). Of the 53 women with a positive AF culture, 13 (25%) had no evidence of HCA, of whom 12 had PPROM and one had preterm labor. The microorganisms that were isolated from the amniotic cavity included *Ureaplasma urealyticum* (n = 38), *Mycoplasma hominis* (n = 30), *Streptococcus viridans* (n = 5), *Streptococcus agalactiae* (n = 2), *Staphylococcus aureus* (n = 2), *Staphylococcus mitis* (n = 1), *Staphylococcus oralis* (n = 1), *Escherichia coli* (n = 1), *Lactobacillus* species (n = 1), unidentified gram-positive cocci (n = 3), unidentified gram-negative rod (n = 1), and unidentified gram-positive rod (n = 1). Polymicrobial invasion was present in 31 cases (58%, 31/53). HCA was present in 36% (36/100) of cases with a negative AF culture and in 75% (40/53) of those with a positive AF culture.

**Fig 1:**
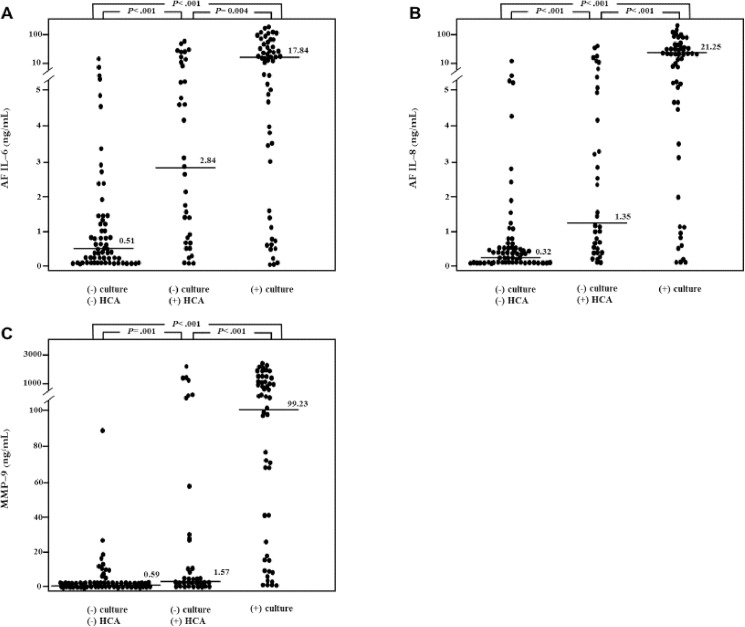
Amniotic Fluid (AF) Interleukin (IL)-6, IL-8, and Metalloproteinase-9 (MMP-9) levels of the study population according to the results of the placental histological examination and AF culture. AF IL-6, IL-8, and MMP-9 levels were lowest in women with a negative AF culture without histologic chorioamnionitis (HCA) (group 1), followed by those with HCA but a negative AF culture (group 2), and highest in those with a positive AF culture (group 3) (AF IL-6: group 1, median, 0.509 ng/mL [range, 0.004–11.825 ng/mL] vs. group 2, median, 2.842 ng/mL [range, 0.047–59.934 ng/mL] vs. group 3, median, 17.839 ng/mL [range, 0.009–104.121 ng/mL]; AF IL-8: group 1, median, 0.318 ng/mL [range, 0.012–11.492 ng/mL] vs. group 2, median, 1.348 ng/mL [range, 0–52.335 ng/mL] vs. group 3, median, 21.253 ng/mL [range, 0.046–113.969 ng/mL]; AF MMP-9: group 1, median, 0.593 ng/mL [range, 0–87.920 ng/mL] vs. group 2, median, 1.572 ng/mL [range, 0–2267.941 ng/mL] vs. group 3, median, 99.232 ng/mL [range, 0–2303.970 ng/mL]; each *P*-value is shown on the graph).

Table 2 shows the clinical characteristics and pregnancy outcomes of the study population according to the results of the placental histological examination and AF culture. Women with HCA but a negative AF culture (group 2) and those with a positive AF culture (group 3) had a significantly lower mean gestational age at amniocentesis and delivery, a higher mean maternal serum CRP level and WBC count, and a higher rate of funisitis than those with a negative AF culture without HCA (group 1). However, no significant differences in these independent parameters were found between the women in group 2 and those in group 3. AF IL-6, IL-8, and MMP-9 levels, and the rate of intra-amniotic inflammation were highest in group 3, followed by group 2, and lowest in group 1 (Fig 1). In terms of AF IL-6 level and the rate of intra-amniotic inflammation, these results were not changed after adjusting for potential confounders such as gestational age at amniocentesis and the state of fetal membranes, showing significant differences among the 3 groups. When compared with the women in group 1, the women in group 3 had significantly more frequent clinical chorioamnionitis, but not those in group 2. However, the mean maternal age and rates of nulliparity, PPROM, antibiotic administration, corticosteroid treatment, and cesarean delivery did not differ among the 3 groups of women.

**Table 2:** Clinical characteristics and pregnancy outcome of the study population according to the results of placental histological examination and amniotic fluid (AF) culture^a^.

Characteristic		Negative AF culture						Positive AF culture		
		HCA negative (group 1; n = 64)	*P* value^b^		HCA positive (group 2; n = 36)	*P* value^c^		Group 3 (n = 53)	*P* value^d^	
			Un-adjusted	Adjusted^e^		Un-adjusted	Adjusted^e^		Un-adjusted	Adjusted^e^
Maternal age (years)		30.8 ± 3.4	.252	.226	31.7 ± 4.4	.737	.854	31.4 ± 3.2	.363	.072
Nulliparity		50% (32)	.423	.549	42% (15)	.871	.819	43% (23)	.476	.389
Membrane status			.021	-		**.007**	-		.586	-
	Intact membranes	48% (31)			72% (26)			43% (23)		
	Preterm PROM	52% (33)			28% (10)			57% (30)		
Gestational age at amniocentesis (weeks)^f^		32.0 ± 3.7	**.001**	-	30.1 ± 3.8	.469	-	29.3 ± 4.3	**< .001**	**-**
Gestational age at delivery (weeks)^f^		32.1 ± 3.7	**.001**	-	30.2 ± 3.8	.462	-	29.4 ± 4.3	**< .001**	**-**
Antibiotics		68% (43)	.321	.651	58% (21)	.019	.321	81% (43)	.115	.424
Corticosteroids		48% (31)	.223	.405	61% (22)	.771	.507	64% (34)	.089	.021
Cesarean delivery		44% (28)	.946	.770	44% (16)	.169	.141	30% (16)	.132	.714
Maternal WBC (10^3^/mm^3^)^g^		10.7 ± 3.7	**.003**	.072	12.6 ± 3.3	.767	.820	12.6 ± 3.8	**.003**	.033
Maternal CRP (mg/dL)^f^		0.6 ± 1.0	**< .001**	.031	1.6 ± 1.8	.201	.139	2.2 ± 2.1	**< .001**	**.001**
AF WBC counts (10^2^/mm^3^)^f^		0.5 ± 2.3	.306	.037	5.4 ± 13.4	**< .001**	.037	32.9 ± 103.4	**< .001**	**.001**
AF IL-6 (ng/ml)^f^		1.4 ± 2.1	**< .001**	**.005**	8.0 ± 0.01	**.004**	**.003**	28.3 ± 0.03	**< .001**	**.001**
AF IL-8 (ng/ml)^f^		1.0 ± 2.0	**< .001**	.032	6.4 ± 12.6	**< .001**	**.001**	30.4 ± 32.2	**< .001**	**< .001**
AF MMP-9 (ng/ml)^f^		3.8 ± 11.8	**.001**	.301	208.8 ± 500.7	**< .001**	**.005**	627.7 ± 734.4	**< .001**	**< .001**
Intraamniotic inflammation^h^		25% (16)	**< .001**	**.002**	67% (24)	.043	**.005**	85% (45)	**< .001**	**< .001**
Funisitis		0% (0)	**.001**	**.008**	22% (8)	.040	.081	43% (23)	**< .001**	**.001**
Clinical chorioamnionitis		2% (1)	> .999	.764	3% (1)	.025	.099	19% (10)	**.002**	.047

AF, amniotic fluid; HCA, histologic chorioamnionitis; PROM, premature rupture of membranes; WBC, white blood cell; CRP, C-reactive protein; IL, interleukin; MMP, matrix metalloproteinase.

Data are mean ± standard deviation, analyzed by Mann-Whitey *U* test among the 3 groups; and % (n), analyzed by χ^2^ test or Fisher’s exact test among the 3 groups.

^a^Significant findings (*p* < 0.0167) after Bonferroni correction are presented in bold letters.

^b^Comparison between group 1 and 2.

^c^Comparison between group 2 and 3.

^d^Comparison between group 3 and 1.

^e^Adjusted for gestational age at amniocentesis and the state of fetal membranes (logistic regression analysis).

^f^*P* < .001, by Kruskal-Wallis analysis of variance test.

^g^*P* < .01, by Kruskal-Wallis analysis of variance test.

^h^Intra-amniotic inflammation was defined as elevated AF levels of IL-6 (≥1.5 ng/mL) and/or IL-8 (≥1.3 ng/mL).

Measures of neonatal outcomes for the 3 groups based on the results of the placental histological examination and AF culture are shown in Table 3. Composite neonatal morbidity was significantly higher among the women in groups 2 and 3 than among those in group 1. However, this was no longer significant after adjusting for confounders caused mainly by the impact of gestational age. The incidence rates of early-onset neonatal sepsis, RDS, BPD, IVH, PVL, NEC, and death showed only slight differences between neonates born to women in groups 1, 2, and 3, except that the incidence rates of RDS in group 3 and BPD in group 2 were significantly increased compared with those in group 1. However, these were also insignificant increases after adjusting for confounders (gestational age and the state of fetal membranes).

**Table 3:** Neonatal outcome of the study population according to the results of the placental histological examination and Amniotic Fluid (AF) culture^a^

Neonatal outcome	Negative AF culture						Positive AF culture		
	HCA negative (group 1; n = 64)	*P* value^b^		HCA positive (group 2; n = 36)	*P* value^c^			*P* value^d^	
		Un-adjusted	Adjusted^e^		Un-adjusted	Adjusted^e^	(Group 3; n = 53)	Un-adjusted	Adjusted^e^
Birthweight (kg)^f^	1.97 ± 0.68	.028	−	1.66 ± 0.67	.353	−	1.51 ± 0.70	**.001**	−
Apgar <7 at 1min^g^	43% (26/60)	.042	.171	66% (21/32)	.616	.279	60% (27/45)	.091	.922
Apgar <7 at 5 min^g^	7% (4/60)	**.004**	.067	31% (10/32)	.694	.995	36% (16/45)	**< .001**	.053
Composite neonatal morbidity^g^	20% (12/60)	**.003**	.217	50% (16/32)	.630	.725	56% (25/45)	**< .001**	.088
EONS^g^	3% (2/60)	.047	.377	16% (5/32)	> .999	.988	16% (7/45)	.036	.457
RDS^g^	13% (8/60)	.018	.710	34% (11/32)	.924	.411	33% (15/45)	**.014**	.925
BPD^h^	8% (5/60)	**.014**	.370	29% (9/31)	.456	.141	21% (9/42)	.059	.363
NEC^h^	5% (3/60)	> .999	.318	7% (2/31)	> .999	.933	7% (3/42)	.688	.505
IVH (grade 2 or more)^h^	2% (1/60)	> .999	.553	3% (1/31)	.387	.262	10% (4/42)	.156	.308
PVL^h^	5% (3/60)	> .999	.584	3% (1/31)	.127	.069	17% (7/42)	.087	.134
Neonatal mortality	0% (0/60)	.355	> .999	3% (1/33)	.634	.676	7% (3/45)	.076	.999

AF, amniotic fluid; HCA, histologic chorioamnionitis; EONS, early onset neonatal sepsis; RDS, respiratory distress syndrome; BPD, bronchopulmonary dysplasia; NEC, necrotizing enterocolitis; IVH, intraventricular hemorrhage; PVL, periventricular leukomalacia.

Data are mean ± standard deviation, analyzed by Mann-Whitey *U* test among the 3 groups; and % (n/N), analyzed by χ^2^ test or Fisher’s exact test among the 3 groups.

^a^Significant findings (*p* < 0.0167) after Bonferroni correction are presented in bold letters.

^b^Comparison between group 1 and 2.

^c^Comparison between group 2 and 3.

^d^Comparison between group 3 and 1.

^e^Adjusted for gestational age at delivery and the state of fetal membranes (logistic regression analysis).

^f^*P* < .01, by Kruskal-Wallis analysis of variance test.

^g^Sixteen infants were excluded from the analysis because they died in utero after amniocentesis (n = 1) or were not actively resuscitated at birth because of extreme prematurity (n = 15) and thus could not be evaluated with respect to the presence or absence of complications.

^h^Based on 133 subjects who survived for at least 30 days after birth.

## Discussion

We show that (1) among women who delivered preterm neonates owing to preterm labor or PPROM, the prevalence of HCA with a negative AF culture was 23% and that (2) similar to pregnant women with positive AF cultures, women with HCA with a negative AF culture are at increased risk of clinical presentation of symptoms and delivery at earlier gestational ages, intra-amniotic inflammation, and prematurity-associated composite neonatal morbidity. These findings underscore the role of HCA, regardless of AF culture results, as a major risk factor of preterm birth and adverse perinatal outcome, and the need to develop novel rapid biomarkers with greater sensitivity, especially noninvasive ones, for detecting early HCA in the preterm setting.

In regard to HCA and MIAC in women who delivered preterm neonates, the prevalence, inverse correlation with gestational age at presentation and birth, and the relationship with adverse outcome are in line with the observations previously reported by our group and other groups [6, 7, 10, 12, 15]. Moreover, in the present study, the frequency of HCA with negative AF culture was 23%, also in keeping with the findings previously reported by Yoon et al. and our group [7, 10]. The most plausible causes of HCA in women with negative AF culture are as follows: (1) early stage of ascending intrauterine infection in which microorganisms reside in the decidua; (2) an extra-amniotic infection; (3) intra-amniotic viral infection; (4) non-infectious cause of placental inflammation; and (5) MIAC that do not grow in standard culture conditions because of a small inoculum size but can be detected by molecular methods such as polymerase chain reaction (PCR). In support of our speculation regarding inoculum size, a previous study by Kacerovsky et al. demonstrated that the presence of HCA in women with PPROM was associated with a higher bacterial load of genital mycoplasma DNA in AF [16]. On the contrary, we found that 25% (13/53) of women with MIAC had no evidence of HCA, of whom 92% (12/13) had PPROM and 8% (1/13) had preterm labor. This is likely to be due to the possibility that microorganisms directly traverse intact membranes or that microbial invasion may have developed in the interval between amniocentesis and delivery, especially by an ascending route through ruptured membranes in cases with PPROM [17].

Several investigators have reported that the incidence of intra-amniotic inflammation (as assessed by a mass-restricted score or glucose level) was significantly higher in patients with HCA than in those without HCA, all of whom delivered within 48 hours of amniocentesis [9, 18]. In accordance with previous reports [9, 18], our results demonstrated that women with HCA who had negative AF culture results showed stronger intra-amniotic inflammatory response (elevated AF IL-6, IL-8, and MMP-9 levels) and a higher rate of intra-amniotic inflammation than those without HCA with negative AF culture. However, with respect to its design, our study is different from previous studies in that women with positive AF culture were excluded from the HCA group for evaluation of the direct effect of HCA per se (not in combination with MIAC) on the inflammatory status of the AF. Potential explanations for our aforementioned observations can be largely similar to the explanations invoked for the presence of HCA in cases with negative AF culture in the previous paragraph. Furthermore, we found that the magnitude of intra-amniotic inflammatory response was greater in the women with positive AF culture than in those with HCA with negative AF culture. This finding is natural, given the fact that the presence of a large number of microbes to the extent of proliferation into the AF by using the standard culture technique may induce more intense cell-mediate immune system activation and cytokine release than the presence of microbes in the chorioamnion in the absence of AF infection or a small number of microbes in the AF [19, 20].

In the literature, although inconsistent, data suggest that HCA may be a risk factor of preterm birth, and neonatal morbidity and mortality, including early onset neonatal sepsis, BPD, IVH, PVL, and cerebral palsy [2, 4, 6]. However, whether such morbidity and mortality are independent of gestational age remains unclear. In a recent report with a relatively large sample size, Lee et al. showed that the higher incidence of neonatal morbidity according to increased stage of HCA or funisitis was due to an earlier gestational age at delivery [21]. Similarly, in terms of the association of MIAC and elevated AF IL-6 levels with neonatal morbidity, Rodriguez-Trujillo et al. and Comb et al. demonstrated that these significant associations disappeared after adjusting for gestational age at delivery [22, 23]. In concurrence to the results of previous studies [21–23], our results showed that HCA with negative AF culture was associated with increased risk of composite neonatal morbidities, but this risk is dependent on gestational age at birth. Collectively, our findings and those of other groups [21–23] suggest that gestational age at delivery may have a more important role in the development of composite neonatal morbidities than exposure to prenatal infection/inflammation.

The present study has several limitations. First, the study was of retrospective nature, potentially leading to selection bias, although most data were collected prospectively. Second, the study population consisted of a heterogeneous status of fetal membranes, including intact or rupture membranes. However, our results remain valid because we derived our results from a multivariable analysis, adjusting for this confounder. Third, the present study used neither placental culture, including the space between chorioamniotic membranes, nor molecular technique (i.e., PCR) to detect microbes or their DNA in AF. Therefore, we could not specifically explain the causes of HCA with negative AF culture. The strength of our study is the relatively large sample size, yielding clear results, given the low frequency of delivery within 48 hours of amniocentesis. Furthermore, it is the first study to examine the effects of HCA with negative AF culture on maternal and neonatal outcomes, and inflammatory status of the AF.

## Conclusion

Our study shows that among the women who delivered preterm neonates, HCA with a negative AF culture was associated with increased risks of preterm birth, intense intra-amniotic inflammatory response, and prematurity-associated composite neonatal morbidity. These risks are similar to the risk posed by positive AF culture. Further studies with large sample sizes are needed to assess the possible effect of HCA per se on the long-term outcomes (e.g., cerebral palsy) of premature, particularly extremely premature, infants.

## Supporting information

S1 File Raw data. (SAV)
